# Digital Citizenship and Professional Digital Competence — Swedish Subject Teacher Education in a Postdigital Era

**DOI:** 10.1007/s42438-022-00291-7

**Published:** 2022-02-17

**Authors:** Alex Örtegren

**Affiliations:** Department of Applied Educational Science, Umeå University, Umeå, Sweden

**Keywords:** Digital citizenship, Professional digital competence, Teacher education, Postdigital, Democratic assignment

## Abstract

Teacher education (TE) is not only about skills and knowledge but also about citizenship formation as student teachers are prepared for the democratic assignment of school. In a postdigital era, blurred boundaries between digital technologies and physical reality place new demands on citizenship, teacher education institutions (TEIs), and teacher educators (TEDs). This paper explores Swedish TEDs’ views of digital citizenship and the professional digital competence (PDC) required for teaching subject student teachers to teach for digital citizenship. Seven TEIs participated and 16 semi-structured interviews were conducted with TEDs teaching a Core Education Subjects module on education and democracy mandatory for all student teachers. TEDs generally believe that the digitalization of society impacts the democratic assignment and addressing this requires specific PDC. Conceptualizations of digital citizenship tend to foreground source criticism as well as ethical, safe, and sound use of digital technologies, and to some degree also (im-)material means of democratic participation. While generally believing that TE should address questions relating to digital citizenship and that TEDs have an important role in this regard, digital technologies are discussed in the module coincidentally and TEDs are unsure to what degree student teachers receive such training. Challenges include lack of time and unclear Degree Objectives. To develop TEDs’ PDC to include questions relating to digital citizenship in their teaching, support is needed through policy and continuous professional development for TEDs, including reviews of course content and program structure. Future TE research needs to explore digital citizenship in the school subject social studies.

## Introduction

Following the horrors of totalitarianism of World War II, the fostering of democratic citizens was made a key pillar of education in Sweden (Ekman 2007) and many other countries (United Nations 1945). Thus, education is not just about skills and knowledge, but also about citizenship formation (Carr and Hartnett 1996; Dahlstedt and Olson 2014; Englund 1986/2005). Teacher education (TE) is a key arena for the government to impact schools (Hanell 2018) and for the realization of the democratic assignment through the work of teacher educators (TEDs) preparing student teachers (Darling-Hammond in Martin and Mulvihill 2017; Raiker and Rautiainen 2020).

In a postdigital era (Jandrić et al. 2018), the embeddedness of digital technologies impacts citizenship through blurred boundaries between technologies, social networks, the physical, the digital (Frau-Meigs et al. 2017), and interpersonal and human–machine relationships (Burbidge et al. 2020). Going beyond Marshall’s (1950) description of the relation between the citizen and the nation state, which was influential in the twentieth century, this impact can be understood in the context of a broadened understanding of citizenship (Yuval-Davis 1997). From such a perspective, citizenship is viewed as something individuals do rather than merely have (van Gunsteren 1998/2018) where the digital is an important dimension (Choi 2016; Jørring et al. 2019; cf. Carretero et al. 2017). Salient examples include increasingly digital civic engagement (Lindgren 2017), post-truth politics and disinformation (Frau-Meigs et al. 2017), and digital surveillance (Colaresi 2020) in conjunction with artificial intelligence (Burbidge et al. 2020).

While digital technologies place new demands on citizenship and by extension on TEDs and teacher education institutions (TEIs) in preparing student teachers for the democratic assignment, demands also stem from teachers’ having to be ready to teach in an era where digital technologies are embedded in schools and society (Starkey 2016, 2020). Recent Swedish policies on the digitalization of K-12 schools and pupils’ development of ‘adequate digital competence’ put pressure on TEDs who are to teach student teachers how to teach with technology (Lindfors et al. 2021). This requires professional digital competence (PDC): profession-based skills, knowledge, and an understanding of digital technologies in educational contexts (Krumsvik 2011; Lund et al. 2014), which TEDs need to prepare student teachers for the fostering of democratic citizens, addressing the demands placed on citizenship in a postdigital era.

In the field of digital citizenship, educational research has largely focused on teachers and pupils in K-12 classrooms while TE has not been studied to the same extent. Examples include TEDs’ and student teachers’ perspectives in relation to specific areas of digital citizenship, such as responsible use of digital technologies (e.g., Gudmundsdottir and Hatlevik 2020; Lindsey 2015), which may be limited in conceptual scope (Heath 2018). This indicates a need for more educational research on digital citizenship (Christensen et al. 2021; Lauricella et al. 2020) and specifically TE research with broader approaches to digital citizenship, also focusing on Sweden. 

Therefore, in a Swedish TE context, the paper seeks to address the following research questions:

- How do TEDs view digital citizenship and the PDC required to teach for digital citizenship?
- What are the potential implications for TEDs’ role in preparing subject student teachers to teach for digital citizenship?

The digitalization of society restructures social life around digital communication and media infrastructures, for instance, physical communication networks, smartphones, and social media platforms (Brennen and Kreiss 2016). Education has witnessed globally an introduction of hardware, applications, and infrastructure, which has impacted teachers’ work (Starkey 2020) and, by extension, TEDs who prepare student teachers for the fostering of citizens in a postdigital era. While the digital tends to be associated with views of digital technologies as a universal solution to a wide array of problems in society past, present, and future (Rahm 2019), the postdigital reflects a critical approach to technology, society, and grand narratives of technological development. It does not break away from the past and still it recognizes that the state of things is not the same: ‘We are increasingly no longer in a world where digital technology and media is separate, virtual, “other” to a “natural” human and social life’ (Jandrić et al. 2018: 893). The digital is an integrated dimension of a totality as digital technologies are ‘composed of material elements that interact with digitally constituted information’ (Fawns 2019: 142). This echoes how digital citizenship must be viewed alongside other conceptualizations of citizenship as ‘it is not a single dimension and/or a suddenly abrupt change in what citizenship means’ (Choi 2016: 589). The postdigital thus highlights a problematizing, multi-faceted understanding of the digital in a society where digital technologies are *embedded* in social, economic, and political contexts (Cramer 2014; Knox 2019). This includes a ‘raising awareness of blurred and messy relationships between physics and biology, old and new media, humanism and posthumanism, knowledge capitalism and bio-informational capitalism’ (Jandrić et al. 2018: 896). In a postdigital era, these blurred and messy relationships mean that digital citizenship is not confined to ‘the digital’ or ‘the online’; rather, it is non-linear and interrelated with the material world (cf. Choi 2016). This paper highlights the demands in a postdigital era placed by digital technologies on citizenship, TEDs, and teachers, supporting critical reflections on technology in education, particularly conceptualizations of digital citizenship, in ways that reflect the embeddedness of digital technologies.

## Conceptualizing Digital Citizenship

In the field of digital citizenship, there is no universally adopted definition (Frau-Meigs et al. 2017; Richardson et al. 2021) and it is interdisciplinary, which is reflected by recent literature reviews of empirical research and theoretical frameworks in social science (e.g., Jæger 2021; Jørring et al. 2019) and education (e.g., Heath 2018; Richardson et al. 2021). Broadly, digital citizenship has been referred to as opportunities and abilities to use technology for participation in society (Lindgren 2017; Mossberger et al. 2007), and conceptualizations typically include skills, knowledge, attitudes, and behaviors.

In education, normative approaches to digital citizenship are common, focusing on ‘the norms of appropriate, responsible behavior with regard to technology use’ (Ribble 2015: 15), such as Ribble and Bailey (2007), International Society for Technology in Education Standards (2019), and James et al. (2021). Normative approaches, particularly Ribble’s (2015), have been criticized for lack of empirical support, inadequate theorization, and depoliticized citizenship (Heath 2018; Mattson 2016; Noula 2019). While Richardson et al. (2021) argue that ethics has received insufficient attention, other studies show that normative approaches are over-represented and call for more critical approaches (Heath 2018; Jørring et al. 2019).

Others have broadened the conceptual scope. For instance, Jones and Mitchell (2016) add civic engagement to digital citizenship, and Choi (2016) adds a critical dimension which is more social justice oriented along with ethics, media and information literacy, and types of online engagement. In Europe, examples include frameworks developed by the Joint Research Centre of the European Commission. The digital competence framework ‘DigComp’ highlights the use of digital technologies for work, learning, and participation (Ferrari 2013), and the subsequent iterations ‘DigComp 2.1’ (Carretero et al. 2017) and ‘DigCompEdu’ for educators (Redecker 2017) accentuate the focus on citizenship and digital technologies. Similarly, in a Council of Europe publication, Frau-Meiggs et al. (2017) highlight engagement and responsible participation while also linking digital citizenship to lifelong learning.

Conceptualizations of digital citizenship can impact education differently as they highlight different aspects. While the examples of conceptualizations above are not exhaustive, recurring elements typically include ethics, skills, and participation and, sometimes, critical approaches.

While not strictly adhering to one definition of digital citizenship, this paper draws inspiration from Choi (2016). Choi incorporates many elements of the conceptualizations above by including ethics, media and information literacy, civic engagement, and critical approaches to digital citizenship. Also, Choi describes digital citizenship as ‘interrelated but non-linear with offline (place-based) civic lives’ (2016: 565), which could reflect the blurredness in a postdigital era between the digital and the physical, social networks, interpersonal and human–machine relationships, and how this impacts digital citizenship.

Ideals of digital citizenship are also present in Swedish and Nordic K-12 curricula. While Sweden does not clearly link digital technologies/competencies to pupils’ ability to influence society, its K-12 curricula clarify the democratic foundation of education and pupils’ ability to act in a complex reality with increasing digitalization. This includes critical and responsible approaches to digital technologies, highlighting the use and understanding of risks related to digital technologies (Christensen et al. 2021). Thus, digital citizenship is present in Swedish K-12 curricula, and national curricula are examples of what TE needs to consider to realize Degree Objectives relating to the democratic assignment (Edling and Liljestrand 2020).

## Conceptualizing PDC in TE

This paper draws conceptual inspiration from Norwegian TE where professional digital competence (PDC) refers to the profession-based skills, knowledge, and understanding of digital technologies of teachers, and by extension TEDs, whose use of digital technologies is different compared to other groups given the context of education (Krumsvik 2011). For example, TEDs appropriate technologies *and* teach student teachers to appropriate technologies for purposeful use in educational contexts (Lund et al. 2014).

Ottestad et al. (2014) describe three dimensions of PDC: (non-subject related) generic digital competence, (subject-related) didactic digital competence, and profession-oriented digital competence which covers other areas of teachers’ work, for example, communicating with parents. PDC is not a fixed concept, and recently transformative agency has been described as a fourth pillar of PDC, which refers to the ability to engage in change efforts when facing challenges in technology-rich contexts (Brevik et al. 2019), such as unanticipated situations relating to technology use that could impact pupils’ citizenship formation. Engen (2019) highlights context sensitivity as an important aspect of PDC, being able to translate skills and competencies to different situations and domains, for example, school subjects, which points to a dynamic understanding of PDC as a mesh of interrelated dimensions and, as Instefjord (2014) suggests, not focused on the mastery of tools but a critical and reflective understanding of digital technologies in knowledge processes.

This paper uses PDC as a conceptual model to explore how conceptualizations of digital citizenship relate to dimensions of TEDs' PDC. This approach echoes Gudmundsdottir and Hatlevik’s (2020) use of PDC to examine TE focusing on digital responsibility, which is often included in conceptualizations of digital citizenship.

## New Demands on PDC

Teachers need to be ready to teach in an era where digital technologies are embedded in schools and society (Starkey 2016, 2020). In Sweden, recent national policies focus on digital competence in education, which are part of a global policy discourse linked to growth and global competition (Almén and Bagga-Gupta 2019; Hanell 2018). These include the government’s national strategy for the digitalization of school (Government Decision I:1 2017), the 2018 revision of national K-12 curricula (cf. Swedish National Agency for Education 2018), and the national action plan #skolDigiplan adopted by the Swedish Association of Local Authorities and Regions (2019). The policy stipulates that Swedish pupils are to develop 'adequate digital competence', which puts pressure on TEDs who are to teach teaching with technology. This includes ensuring that student teachers graduate with PDC to help their future pupils develop digital competence in line with Swedish K-12 policy (Lindfors et al. 2021).

In addressing these policy changes, several challenges have been identified, such as lack of TE policy support and TEDs’ need for professional development (Lindfors et al. 2021), but also that the meaning of adequate digital competence is unclear (Fransson et al. 2018), which may have consequences. For instance, it may impact pupils’ citizenship formation when interpreted differently in schools (Olofsson et al. 2019), which reflects the more holistic approach to digital competence in the Nordic countries, going beyond technical skills to include, for instance, critical reflections on the use of digital technologies in relation to issues in wider society (Godhe 2019; Krumsvik 2011; McGarr and McDonagh 2019). Similarly, a lack of conceptual clarity may have an impact throughout TE, affecting student teachers’ PDC if interpreted differently at TEIs, or TEDs’ PDC if interpreted differently, for instance, in professional development programs, foregrounding certain meanings while overlooking others.

## Previous Research

### Preparing Student Teachers for the Democratic Assignment

Previous studies have identified several challenges when preparing student teachers for the democratic assignment. Although the democratic assignment concerns everybody in school, student teachers sometimes regard the teaching of citizenship as unrelated to their school subject and thus other teachers’ responsibility (Carr 2012). Another example is not adequately considering future pupils’ social background, especially socially disadvantaged pupils, whereby preparation for the democratic assignment becomes disconnected from the communities where future teachers will work (Zeichner 2020).

Different conceptualizations of democracy can also impact future teachers’ engagement with the democratic assignment (Bernmark-Ottosson 2005; Carr 2012; Eriksen 2018). Conceptualizations are often discussed using contrasts such as *thick-thin* or *broad-narrow* to illustrate the differences between broad notions of democracy, involving, for instance, equality and people’s everyday actions, and narrow notions focusing on institutional democratic practices (Edling and Liljestrand 2018). Student teachers’ conceptualizations of democracy tend to be narrow with apolitical notions of democratic work, and therefore they need TEDs’ support to develop skills, knowledge, and a deeper understanding of democratic work (Carr 2012; Zyngier 2012). This includes highlighting how different approaches to democracy have implications when teaching for democracy (Edling and Liljestrand 2018; Eriksen 2018).

Broad and narrow notions of democracy are not to be viewed as dualistic opposites (Edling and Liljestrand 2020) but complementary; knowledge of institutional democratic practices contributes to the foundation for democratic work, and schools play a key role in providing pupils with opportunities to experience and develop skills and knowledge of democracy. To this end, teachers need to make pedagogically and theoretically informed choices (Stray and Sætra 2018), which further highlights the importance of how TEDs prepare student teachers for the democratic assignment. However, in the studies above, it is unclear how TEDs can support student teachers’ development of skills, knowledge, and understanding of democracy in relation to digital technologies.

### Digital Citizenship

Educational research on digital citizenship has largely focused on K-12. Some studies have focused on K-12 curricula in Sweden, the Nordic countries (Christensen et al. 2021), and elsewhere (Davis 2020; Kingsmill 2016), or specific digital citizenship curricula such as Ribble’s nine elements of digital citizenship (Mattson 2016; Noula 2019). Studies have also focused on pupils, primarily grounded in normative approaches to digital citizenship, for instance, in intervention studies (Tapingkae et al. 2020; Vlaanderen et al. 2020) or measuring impact of teaching (Bickham et al. 2021; Jones and Mitchell 2016), sometimes including school leaders and teachers (Kingsmill 2016). Studies have also focused on teachers, often using surveys, which have shown a tendency toward teaching aspects of digital citizenship perceived as positive for character development (Lauricella et al. 2020) and a strong influence from years of teaching, use of social networking services, and perceived self-efficacy (Choi et al. 2018), which may be high while interviews reveal an inability to teach for digital citizenship (Szakasits 2018).

Similar to research on PDC in education as shown below, TE has received less attention than K-12. Studies have often focused on responsible use of digital technologies. One example is Gudmundsdottir and Hatlevik’s (2020) interviews with Norwegian student teachers about their perceived preparation prior to and during placement. Student teachers were uncertain about privacy and copyright issues, and even if they knew how to search for information, convenience played a large role. A challenge during placement was the focus on technical aspects ‘rather than pedagogical or responsible ways’ of technology use (Gudmundsdottir and Hatlevik 2020: 52). Another example is Lindsey’s (2015) American study of a professional development program which includes TEDs and support staff. Survey data, observations, interviews, and memos show that the program promoted TEDs’ PDC in relation to digital citizenship in ways that made student teachers more determined to teach for digital citizenship. While these are important contributions to the understanding of digital citizenship in TE, their conceptual scope covers only certain aspects of digital citizenship (Heath 2018; Jørring et al. 2019), which indicates a need for TE research open to approaches beyond the normative strand of digital citizenship.

### TEDs’ PDC and Development of Student Teachers’ PDC

While a large body of research has focused on K-12 teachers’ PDC and use of digital technologies, fewer studies have focused on TEDs’ PDC and the task of teaching student teachers how to teach with digital technologies (Uertz et al. 2018). Previous studies have highlighted TEDs’ need for continuous professional development in PDC (Amhag et al. 2019; Instefjord and Munthe 2017; Lindfors et al. 2021). As TEDs’ PDC correlates with their perceived efficacy (Instefjord and Munthe 2017; Herro et al. 2021), TEDs need opportunities to see the value added by using digital technologies demonstrated by experienced teachers acting as role models (Amhag et al. 2019; Gudmundsdottir and Hatlevik 2018; Lindfors et al. 2021; Tondeur et al. 2012). Also, teachers’ beliefs and attitudes are important (Scherer et al. 2018; Tondeur et al. 2017; Uertz et al. 2018), and TEDs need to provide student teachers with continuous feedback to foster attitudes conducive to PDC development (Baran et al. 2019). TEDs’ task of promoting student teachers’ PDC becomes even more important considering how placement periods tend to focus on technical rather than pedagogical aspects of using digital technologies (Gudmundsdottir and Hatlevik 2020). Otherwise, student teachers risk graduating without the necessary skills and knowledge to help future pupils develop PDC in line with policy.

To further promote TEDs’ development of PDC, support through TE policy is important (Lindfors et al. 2021; Miguel-Revilla et al. 2020) and, likewise, institutional support has been highlighted as necessary (Lindfors et al. 2021; Roumbanis Viberg et al. 2021) although insufficient in explaining what influences teachers’ PDC (Instefjord and Munthe 2017). Even if support is provided, TEDs who perceive themselves as lacking skills and opportunities to engage in professional development can avoid addressing digital technologies in their teaching by relying on their work community whereby a TED can feel less responsible for the consequences of opting out while maintaining a high sense of agency. Hence, the skills, knowledge, and approach of the individual TED can impact student teachers’ PDC development (Roumbanis Viberg et al. 2021).

While previous research on TEDs’ PDC has highlighted among others the role of support through continuous professional development and policy, beliefs and attitudes, and role models, there is a need for more research (Lindfors et al. 2021; Starkey 2020), particularly on TEDs’ competencies for teaching how to teach with digital technologies (Uertz et al. 2018).

### Summarizing Possible Directions for Future Studies

Educational research has largely focused on digital citizenship in K-12 while TE is less explored. Studies have often focused on normative approaches to digital citizenship, and broader conceptual focus is necessary (Heath 2018). While directed efforts such as professional development programs can impact skills, knowledge, and attitudes among TEDs and student teachers (Lindsey 2015), more research is needed on digital citizenship and teaching for digital citizenship (Christensen et al. 2021; Lauricella et al. 2020). Similarly, more research has been called for on TEDs’ PDC (Uertz et al. 2018), which has identified a need for support from institutions and national TE policy. This paper responds to calls for research on digital citizenship, including broader conceptual scope, TEDs’ PDC and digital citizenship, and studies on democratic education focusing on individuals and context in an increasingly digitalized world (Swedish Research Council 2019).

## Method

### The Democratic Assignment in Swedish Subject TE

The subject-teacher TE track in Sweden prepares students for teaching the last 3 years of compulsory school and upper secondary school (ages 13–19), which requires a Degree of Master of Arts/Science at the second-cycle level (4 to 5.5 years of full-time study, 240–330 ECTS). Degree Objectives are essentially the same with only slightly higher requirements for upper secondary school teachers and they belong to the same TE track, which provides the rationale for combining these in this paper. Studies include two subjects, subject didactics, placement, and Core Education Subjects (Sw. *Utbildningsvetenskaplig kärna*; 1 year of full-time study, 60 ECTS). Core Education Subjects provide central and generic knowledge necessary for teachers, for instance, learning, assessment, and the democratic assignment. All student teachers are to ‘communicate and instil core educational values, including human rights and the fundamental democratic values’ (SFS 1993:100; cf. SFS 2010:800) as the democratic assignment is not confined to certain school subjects (Englund 1986/2005).

National policy regulates Swedish TE through objectives expressed in broad ways that need to be interpreted by TEIs when designing programs and courses (Edling and Liljestrand 2020). Without fully standardizing Core Education Subjects, the last large TE reform of 2011 (Government Bill 2009/10:89) increased regulation of knowledge, skills, and attitudes that student teachers are to acquire, but ultimately TEIs interpret objectives and design their courses. This means that the democratic assignment can be addressed in various areas of TE (Åstrand 2020); however, it typically takes the center stage in the first semester of Core Education Subjects where Student Learning Outcomes refer explicitly to the democratic assignment (e.g., explain the core values of education), often as a course or a course module (5 weeks, 7.5 ECTS), which in this paper is referred to as the Education and Democracy (E&D) module.

### Data Collection

#### Selection

In Sweden, 25 TEIs prepare subject teachers for compulsory school and, together with two more TEIs, they also prepare subject teachers for upper secondary school (27 in total). Five are located at higher education institutions that specialize in a certain field or attract students mainly in other fields and, with one exception, they offer a limited number of program orientations (≤ 2), focusing on art, crafts, music, physical education, and health. While three of these have numbers of enrolled subject student teachers comparable to other institutions, two were excluded because of size with five or fewer new enrollments in the 2018/2019 academic year (Swedish Higher Education Authority 2020, 2021). Contact requests were sent to seven TEIs of various age and size spread geographically across Sweden.

A purposive sampling approach was used (cf. Cohen et al. 2018) to contact TEDs teaching the democratic assignment in the E&D module in the first Core Education Subjects semester. Gatekeepers, such as the director of studies, provided contact details by e-mail. The number of TEDs involved in the E&D module varied as did their degree of involvement, which in some cases was peripheral (e.g., introducing students to the learning management system) and thus warranted exclusion. 17 potential informants were identified of whom one declined to participate. From each TEI, up to three TEDs were interviewed. At the time of the study, the number of TEDs was limited at three TEIs as fewer were involved, matched the profile, or accepted to be interviewed.

#### Participants

On average, the 16 TEDs had taught TE for 13 years (7–29 years) and the E&D module for 6.5 years (1–10 years). 14 TEDs had taught the module before the 2018 revision of the Swedish national K-12 curricula which introduced new stipulations on digital competence. At six out of seven TEIs, one TED was responsible for the module or shared this responsibility with a colleague. Also, at three TEIs, one department was responsible for the E&D module whereas at the other four, the responsibility was shared with other departments. The majority held positions as senior lecturers and three were associate professors or full-time professors. Two TEDs did not have a PhD but had extensive experience teaching TE. Nine TEDs had a PhD or a master’s degree in education or had completed TE before doing a PhD in another field while seven had a background outside of education and combined teaching TE with work in other fields.

#### Data Description

To develop a thorough understanding of how TEDs view digital citizenship, the PDC required to teach for digital citizenship, and their role in teaching for digital citizenship, semi-structured interviews (cf. Brinkmann and Kvale 2015) were conducted with 16 TEDs between February and April of 2021. E-mails were sent beforehand to the participants, including an information sheet and a written consent form, after which video meetings on Zoom were scheduled. The interviews involved probes and follow-up questions similar to in-depth interviewing (cf. Cohen et al. 2018), for example, ‘What could …, do you think?,’ ‘What is your idea of…?,’ and ‘How do you experience…?,’ including clarifying summaries during the interviews. The interviews lasted between 50 and 80 min (16.3 h in total) and were recorded digitally and transcribed verbatim (238 pages) to capture the dynamics of the dialogue, such as emphases, pauses, and sudden turns. Digressions from the research questions were summarized within brackets. Participants received encrypted transcription copies for approval. A few minor changes were made upon request which were subsequently approved by the participants (e.g., summarizing dialogue to reduce identifiability).

Prior research discussed above provided the basis for an interview guide which provided consistency across three themes: (1) the digitalization of society, (2) the E&D module, and (3) digital citizenship, digital competence, and the potential role of TEDs in preparing subject student teachers to teach for digital citizenship. Also included were background questions (e.g., age, experience, current role in teaching TE and the E&D module, views of technology).

Moreover, course plans were collected, which are control instruments that TEDs need to consider, and study guides, which show how TEDs plan the teaching of the E&D module. These documents contribute to contextualizing the interviews. Some TEDs provided additional materials (e.g., PowerPoint presentations) but these were not included in the analysis. This study complies with the ethical standards specified by the Swedish Research Council (2017) concerning information to participants, consent, confidentiality, and the use of research data.

### Data Analysis

Transcriptions and course documents were imported and categorized in NVivo Release 1.5.1 (QSR International). Although computer software does not necessarily strengthen the quality of the analysis (Flick 2018), NVivo facilitated, for instance, text queries and organizing the data. The researcher initially queried the course documents using search tags, for example, ‘digital,’ focusing on writings related to PDC or digital technologies and possible links to the democratic assignment. To ensure the relevance of the search, documents were read manually after which notes were added and compiled into summaries. Also within NVivo, the researcher performed a reflexive thematic analysis on the interview data. While some schools of thematic analysis are more grounded in post-positivist thinking with pre-determined themes and replicability, reflexive thematic analysis foregrounds researcher subjectivity and self-reflexivity. Thus, meaning-based themes are *generated* rather than discovered through iterative reading and coding (Braun et al. 2019).

Initially, the transcriptions were read, and notes were added in an overview document together with data-linked memo summaries for each TED within NVivo. This phase involved repeated close-readings and adjustment of summaries. Iterative phases of deductive coding followed based on the interview guide themes (e.g., digital citizenship — source criticism). 73 codes were merged into 29 codes and grouped together based on similarity which formed subthemes (e.g., conceptualizations of digital citizenship), which then formed two main themes (see Table 1): support needed to address digital citizenship (four subthemes, 19 codes) and TEDs do not address the democratic assignment in relation to digital technologies (three subthemes, ten codes).

**Table 1:** Example of the coding process

Theme	Subtheme	Codes	Quotes
Support needed to address digital citizenship	TEDs conceptualize digital citizenship differently	Source criticism; ethical, safe, and sound use of digital technologies; (im-)material means for democratic participation	‘it [digitalization] places also other demands because of, well, what should we say … Yes, there’s so much! … That there’s so much information that you have to learn to sift in another way’ ‘not to use [digital] technologies in a way that violates anyone’ ‘you also have more of a possibility not just to find out what is being said on different websites but you can also participate there’

## Results

TEDs in this study frequently refer to the Covid-19 pandemic as a catalyst for the use of digital technologies which has highlighted advantages and, often with an emphasis, disadvantages. While possibly contributing to a pre-pandemic trend of digital exclusion where government information and services become harder to access for the elderly and immigrants, the pandemic and the embeddedness of digital technologies have also provided fertile ground for the spread of disinformation, filter bubbles, and polarization. In discourses in education and wider society, TEDs sometimes perceive traces of technological determinism where technology must be embraced. Some believe that the digitalization of society ‘has gone too far’ and ‘needs be ruled by humans’ while others highlight the advantages and that some negative consequences are inevitable. As a process, the digitalization of society is described either as a constitutive interplay between humans and technology or the result of human activities within societal structures where technology plays only a secondary role. Regardless, boundaries are becoming increasingly blurred between the online and the offline, and what was once virtual has become as ‘real’ as the analog. Digital technologies have become ‘naturalized in our way of thinking’ and impossible to separate out: ‘We don’t reflect on it. It’s just there.’

At the TEIs where these TEDs work, course plans and study guides governing the E&D module indicate few instances where digital citizenship is addressed. At four TEIs, digital technologies are mentioned in relation to availability of study materials, examinations, or evaluations, often in the context of Covid-19 campus restrictions, while three TEIs mention presentation and communication skills with occasional examples of documentation in school and source criticism. Four TEIs address the democratic assignment in relation to specific areas, for instance, human rights. These are generally implicit and reflected by reading lists and time allocation, which is confirmed by TEDs who view this as necessary given the time frame and the subject matter. Hence, there are no explicit links between the democratic assignment and digital technologies or PDC in the course documents.

The descriptions above provide the context for the two main themes: (1) TEDs need support to address questions relating to digital citizenship and (2) TEDs do not address the democratic assignment in relation to digital technologies in the E&D module. These themes contribute to the understanding of TEDs’ views of digital citizenship in relation to the democratic assignment in Swedish subject TE.

### Support Needed to Address Digital Citizenship

TEDs need support to address digital citizenship, which can be linked to four subthemes. While believing that the digitalization of society impacts the democratic assignment, TEDs have different views of how digital citizenship can be conceptualized and their role in teaching to teach for digital citizenship. To include questions relating to digital citizenship in their teaching, TEDs need support from their institutions and through TE policy.

### TE Should Address the Democratic Assignment in Relation to Digitalization of Society

TEDs generally believe that the digitalization of society impacts the democratic assignment and that this needs to be reflected by TE, which indicates a need for PDC to address such questions. Many emphasize how schools need to keep up with societal change but lag behind as fast-paced technological development makes it difficult to keep up with the changing world of pupils. There is also an orientation toward the future where TEDs highlight how student teachers’ future pupils will live their lives and act as citizens increasingly more through digital media, which has implications for how TEIs prepare student teachers. As expressed by one TED, ‘education and society, they cannot be disconnected, they are so deeply connected so if there is a societal change in any way – in this case digitalization – school has to address the changing conditions, the new demands, and so does teacher education, too.’ Some TEDs add that while it might not be possible to devote a specific part of the E&D module to questions relating to the digitalization of society, it would be beneficial if such questions were addressed in TE generally, which points to a need for PDC development throughout TE.

TEDs also believe that TEIs lag behind when it comes to the digitalization of society and its impact on the democratic assignment. Schools and TEIs are described as slow in responding to changes in society generally while technological development is fast. The lag could also be because of TEDs’ assumptions regarding student teachers’ PDC. As students tend to be younger than TEDs, some may doubt whether they have anything to teach student teachers about digital technologies in relation to societal change and therefore choose not to address such questions. This is reflected by descriptions of how TEDs and student teachers contribute with different types of knowledge during lessons: ‘[T]hey know this [digital technologies] much better than I do, so I tell them, what can you contribute with? … I mean, it’s their world and they think it’s hard to enter the *teacher* world.’ Thus, it becomes the student teachers’ task to contribute with skills and knowledge rather than TEDs when it comes to PDC.

Not all TEDs agree that TE lags behind when it comes to the democratic assignment and digital technologies. TEDs at two TEIs argue that ‘it [digital technologies] is there the whole time, there are examples in all parts of the course’ even if there is no lecture or materials devoted to these questions. A minority is undecided or skeptical as to the degree to which the digitalization of society impacts the democratic assignment. For instance, although agreeing that digitalization may impact the democratic assignment, so do other social phenomena of which some could be more important to address in the E&D module.

A small number of TEDs also describe how the digitalization of society impacts the compensatory role of school and, in turn, the democratic assignment, which requires PDC to help *all* pupils develop skills to use digital technologies. Current discourse in education regarding the digitalization of society and its entailing demands is ‘problematic’ as some pupils do not have physical access to digital technologies, such as sufficient bandwidth or a device fit for use, which TEDs suggest has become evident during the pandemic. Also, it is increasingly more important that pupils develop the necessary skills to use digital technologies, and some TEDs relate this to social justice and the compensatory role of school to support disadvantaged pupils and pupils with special needs. One TED argues however that social justice must be understood not in relation to digital technologies but primarily other developments in society, such as school segregation.

### Digital Citizenship Conceptualized Differently

TEDs’ conceptualizations of digital citizenship can be grouped into three categories of which the first two are more common: (a) source criticism; (b) ethical, safe, and sound use of digital technologies; and (c) material and immaterial means for democratic participation. Each category entails teaching for digital citizenship in different ways with different PDC requirements. Moreover, these categories are not exclusive; a TED may conceptualize digital citizenship in ways that reflect several categories.

Highlighted by 11 TEDs, source criticism is the most common conceptualization of digital citizenship. The PDC required involves searching for, selecting, and assessing sources of information based on relevance and value in relation to the purpose of the search. It requires being able to understand how data and algorithms affect the ways in which information is displayed, spread, and the potential consequences thereof. TEDs often mention disinformation, for instance, on social media, and how new abundances of information and rapid communication streams require a new type of source criticism. Fact-checking and sifting through information has become more difficult, which makes it harder to assess sources and their potential value.

Almost as common is the conceptualization of digital citizenship as ethical, safe, and sound use of digital technologies. The PDC required concerns ethical deliberation in the treatment of others, such as cyber-bullying. It includes duties, such as obeying laws and regulations, and being able to communicate and process information as citizens because interaction with the nation state increasingly involves digital technologies. Moreover, this conceptualization concerns being able to reduce the risk of exposure to inappropriate or dangerous content, such as pornography. TEDs also highlight the sound use of digital technologies, referring to screen time and the ability to ‘switch off.’

Less common is the conceptualization of digital citizenship as material and immaterial means for democratic participation. TEDs highlight a participatory dimension of citizenship where digital citizens have the material means, such as a computer or a cell phone, and the skills and knowledge to participate, for instance, on websites or using social media for political mobilization. Immaterial means can also include digital literacy and understanding how data can be used for different purposes in relation to citizens, for example, by political regimes and powerful corporations.

### Views Vary on Teaching to Teach for Digital Citizenship

Most TEDs believe that they have an important role in teaching how to teach for digital citizenship and that student teachers need to start reflecting on these questions. Half of the TEDs argue that student teachers regardless of subject specialization need skills and knowledge in this area and that questions relating to digital citizenship ought to be included in Core Education Subjects, which indicate a certain need for PDC among TEDs teaching the E&D module. Some also suggest that questions relating to digital citizenship could be addressed in all student teachers’ subject studies. Others point to social studies TE as a minimum (‘at least social studies teachers need to have received training in this’), or because these questions are best addressed in social studies TE given that the subject matter includes democracy, which would entail that TEDs teaching subject didactics require PDC to address digital citizenship.

Two small groups differ from the majority in how they view their role in teaching how to teach for digital citizenship, and they do this in ways that suggest PDC is not required. The first group argues that teaching the democratic assignment and addressing digital citizenship requires skills and knowledge that are not specific to digital technologies. The digital is but a medium and the impact of the Internet must not be exaggerated. In this view, the democratic assignment involves ‘basic components’ which are ‘connected to values, abilities, and knowledge … And then this [digital technologies] come as a type of, well, spice added to the original dough.’ What this ‘dough’ requires is extensive knowledge of democracy, for instance, as expressed by the Swedish Constitution, or an ability to reflect on one’s teaching and the activities that take place in the context of teaching. A second group, whose work is mainly in other fields than TE, expresses doubts as to whether they are expected to address digital technologies at all in the E&D module as they contribute in other ways given their background, or they have never worked at a school and thus have never reflected on questions relating to digital citizenship. This suggests that it is TEDs with a background in education that need PDC to address questions relating to digital citizenship.

### Local and National Support

To include questions relating to digital citizenship in their teaching, certain PDC seems needed. TEDs express a need for specific knowledge of digital technologies and digital citizenship, which requires professional development. The majority believe that this would help them make student teachers better prepared for their future careers. Teaching student teachers how to teach for digital citizenship is viewed as important as it may impact pupils’ democratic participation in the future, but ‘this is not a quick fix that you get by one’s coming by and giving a lecture.’ Hence, time allocated for professional development is highlighted as important. Although emphasizing that questions relating to digital citizenship are important, some TEDs suggest that another type of professional development may need to be prioritized first to ensure ‘that they [TEDs] actually focus on TE,’ which indicates challenges in TE not specific to digital citizenship or PDC.

To teach teaching for digital citizenship also seems to require a review of course content, program structure, and Degree Objectives. TE is ‘packed,’ and Degree Objectives in the Higher Education Ordinance emphasize other areas and not the democratic assignment. While believing that the digitalization of society impacts the democratic assignment, questions relating to digital citizenship will not be addressed unless clarified by the Degree Objectives:

Just by reading the Higher Education Ordinance you realize how many things are supposed to be there, everything from the history of education to theories of learning, didactics, curriculum theory, where does this … digitalization … come in? And the way it reads now, it is not a core content in the Higher Education Ordinance and thus it will not appear in the courses.

### TEDs Do Not Address the Democratic Assignment in Relation to Digital Technologies

Reasons why the democratic assignment is not addressed in relation to digital technologies in the E&D module can be linked to three subthemes. These relate to conditions for teaching the module, teaching priorities, and how TEDs address the democratic assignment but not explicitly in relation to digital technologies.

### Democratic Assignment Not Addressed in Relation to Digital Technologies

TEDs generally describe instances of the E&D module where digital technologies are not addressed, let alone in relation to the democratic assignment. Often mentioned are the Degree Objectives in the Higher Education Ordinance, which is described as an important control instrument that does not link, at least explicitly, the democratic assignment and digital technologies. Also, because objectives are broad in scope, there is neither time nor incentive to address the democratic assignment in relation to digital technologies unless such links are clarified. Three TEDs with a background outside of education are uncertain as to whether they are expected to address digital technologies in the E&D module, which is the case at TEIs where module responsibility is shared across departments.

Some TEDs believe that digital technologies are addressed in the E&D module, but this seems to be either coincidental or not explicit. Student teachers might ask questions or make comments, for example, regarding experiences from placement periods, but not all student groups initiate such discussions. Some TEDs maintain that they address digital technologies but not explicitly, such as a devoted lesson or PowerPoint slide. This is reflected by one TED who claims that colleagues believe that the democratic assignment is impacted by digital technologies but that they ‘perhaps do not explicitly mark it out as an objective on the agenda.’

Some speculate that subject didactics, particularly social studies didactics, might address questions relating to digital citizenship. This is often accompanied by some uncertainty expressed through wording such as ‘probably’ or ‘I think,’ or reservations such as ‘I’m not an expert [regarding what other departments do]’ and ‘I don’t know the details when it comes to different subjects.’ In contrast, TEDs with a seemingly strong identification as teachers of subjects suggest that it is in fact those teaching general TE courses that tend to address digital technologies. Such discussions are thought to be about social relationships and cyberbullying, which are treated as a specific topic with its designated reading at some of the TEIs.

### Program Contents and Local Organization Affect Teaching Conditions

Conditions for teaching the E&D module appear to be unfavorable. Challenges include subject matter and allotted time, which are described as general challenges in TE, and one TED believes that they merely ‘scratch the surface’ and that student teachers feel the same. There is a fear that important aspects of the E&D module are not addressed adequately, and particularly troubling to some is the feeling that the fostering of democratic citizens receives little attention in comparison to other subject matter covered in the module. Also, at some TEIs, the module extends over the winter holidays, and as student teachers may not be on campus for 2 out of 5 weeks, the module ‘essentially has to be taught in 3 weeks’ of which the last week is a take-home exam.

Also affecting the conditions for teaching is local module responsibility. At some TEIs, one department is responsible for planning and teaching the E&D module while elsewhere, several departments share the responsibility or collaborate. TEDs are skeptical of how course responsibility is distributed, which is reflected by descriptions of local TEI umbrella organizations that from time to time welcome all departments to present proposals in the hope of ‘winning’ the responsibility of TE courses and thus secure funding and staff. TEDs worry that this course distribution system may cause important aspects of TE to be overlooked. At one of these TEIs, the situation is described as a ‘free-for-all’ where the Department of Education has little influence as other departments have ‘won’ courses in Core Education Subjects.

### Priorities When Teaching the Democratic Assignment

Given the conditions described above, TEDs highlight different priorities in relation to the teaching of the democratic assignment in the E&D module. Student teachers have varying degrees of knowledge and understanding of democracy. Some are too consensus-oriented, or do not reflect sufficiently on their role as teachers in relation to the democratic assignment, or assume that education takes place in democratic forms without reflecting on how and if that is the case.

Therefore, TEDs’ teaching priorities tend to involve meanings and problematizations of democracy in relation to the democratic assignment. The purpose is for student teachers to develop knowledge of democracy and a critical understanding of national K-12 curricula, including how the democratic assignment becomes manifest in education. To this end, some TEDs draw on democratic theory, such as direct democracy, representative democracy, and deliberative democracy. Although not exclusively, dissecting what democracy means in broad terms tends to be expressed by TEDs with a background outside of education.

While Student Learning Outcomes are explicitly linked to democracy in the module, a small number of TEDs emphasize that questions related to the democratic assignment are distributed across Core Education Subjects. For example, when asked about the democratic assignment, one TED asks what part of Core Education Subjects is referred to, and another, as if in response, claims that this depends on how the democratic assignment is defined. This echoes TEDs’ speculations above that questions relating to the democratic assignment may be addressed elsewhere in TE.

## Discussion and Conclusions

The purpose of this paper was to explore TEDs’ views of digital citizenship and the PDC required to teach for digital citizenship, and potential implications for TEDs’ role in preparing subject student teachers to teach for digital citizenship. This paper highlights several implications. The majority believe that the digitalization of society impacts the democratic assignment and how TE prepares student teachers, but TEDs view digital citizenship differently, if at all. Common conceptualizations include source criticism and ethical, safe, and sound use of digital technologies while some also highlight (im-)material means of democratic participation.

In light of Choi’s (2016) paper from which inspiration is drawn, the conceptualizations above give rise to several questions, such as to what degree these reflect the embeddedness of digital technologies in a postdigital era. One example is the framing of norms and civic engagement in ways that potentially position the digital as different (e.g., ‘ethics online,’ ‘participation on the web’) while digital citizenship in a postdigital era may transcend such divisions. This example of framing is also interesting considering TEDs’ descriptions of how digital technologies have become ‘naturalized in our way of thinking,’ highlighting blurred boundaries between technology, the digital, the physical, and the social.

Other questions concern conceptual scope, for instance, the norms that inform ethical, safe, and sound use of digital technologies, and the potential implications for citizenship formation (cf. Mattson 2016; Noula 2019). While responsible use and participation may be important endeavors in developing digital citizenship, an essential question is to what extent these conceptualizations sufficiently address post-truth politics, disinformation, and the role of data and algorithms in a postdigital era, to mention a few examples. Also, there are TEDs for whom the digitalization of society does not impact the teaching for citizenship and, by extension, the preparation of student teachers to teach for citizenship. A similar question, then, is to what extent the impact of digital technologies on citizenship can remain unaddressed if the goal is to prepare student teachers for the democratic assignment in a postdigital era.

The conceptualizations in this paper are not to be viewed as mutually exclusive but rather as potential sources of configurations of digital citizenship, mirroring the ambiguity of digital citizenship (Jones and Mitchell 2016) which in other national contexts has led to calls for policy support (Davis 2020; Kingsmill 2016). Without clear policy, there is a risk that certain aspects are overemphasized or overlooked. For instance, Davis (2020) has shown that normative approaches to digital citizenship are common where no central guidelines are provided, which may explain TEDs’ focus on ethical, safe, and sound use of digital technologies in this paper. A lack of policy support could thus impact equivalence within and across TEIs, affecting how TEDs prepare student teachers for the democratic assignment and, in turn, future K-12 pupils’ citizenship formation (cf. Olofsson et al. 2019). Moreover, if key concepts are unclear, this can impact student teachers’ willingness to engage with theory and their future work in schools (Puustinen et al. 2018).

Therefore, TE policy is needed to put questions relating to digital citizenship on the agenda and support TEIs’ interpretation of TE policy. For instance, Degree Objectives could clearly highlight democratic work and digital technologies as connected. To include questions relating to digital citizenship in TEDs’ teaching, this paper echoes previous calls for TE policy to support TEDs’ PDC (Lindfors et al. 2021; Miguel-Revilla et al. 2020). Increased policy support raises other questions, however, such as who is to be responsible for addressing questions relating to digital citizenship in TE and how digital citizenship is conceptualized.

Different conceptualizations also require different PDC. One way of highlighting this is by making digital citizenship a dimension of PDC or an aspect of current PDC dimensions (cf. Ottestad et al. 2014; Brevik et al. 2019), which could support TEIs in ensuring that TEDs have PDC to include questions relating to digital citizenship in their teaching. For instance, focusing on source criticism in relation to digital technologies may require different skills and knowledge than norms guiding responsible use. Again, this reflects the need for policy support, which has been done, for instance, in Norwegian TE where the *Professional Digital Competence Framework* (Kelentrić et al. 2017) covers several aspects of digital citizenship, such as ethics and participation, even if the term digital citizenship appears only in references to other frameworks. Moreover, previous studies have highlighted TEDs’ need for continuous professional development in PDC and digital technologies (Amhag et al. 2019; Instefjord and Munthe 2017; Lindfors et al. 2021). This paper echoes this need in relation to digital citizenship as TEDs express a need for specific knowledge that requires professional development facilitated by resource allocation, primarily time, indicating the key role of TEIs in this context.

As the democratic assignment concerns everybody at school, the mandatory Core Education Subjects can contribute toward the preparation of all new teachers for democratic work in an era where digital technologies are embedded in schools and society (cf. Starkey 2016, 2020). This paper shows that where Student Learning Outcomes most explicitly relate to democracy, course documents and interviews with TEDs indicate few opportunities for student teachers to develop PDC to include questions relating to digital citizenship in a postdigital era. This is not to say that this should be the case, but the question is where else, nor is this an attempt to blame TEDs for not addressing digital technologies or PDC in the module. TEDs need to support student teachers to develop skills, knowledge, and a broad understanding of democracy (Carr 2012; Edling and Liljestrand 2018; Zyngier 2012). While TEDs believe they have an important role in teaching student teachers to teach for digital citizenship, current conditions for teaching the E&D module make other priorities perceived as necessary, notably because of a lack of time which is a general problem when it comes to democratic work in European TE (Raiker and Rautiainen 2020). Only a minority claims to address the democratic assignment in relation to digital technologies, but if this is the case, further systematization is needed. A starting point could be local TEI reviews of course content and program structure supported through time and resource allocation to ensure that questions relating to digital citizenship are addressed.

Furthermore, in the Swedish context, the E&D module often coincides with placement. This could be a route toward strengthening student teachers’ PDC in relation to digital citizenship and broaden their understanding of democracy in ways that reflect the embeddedness of digital technologies in a postdigital era (cf. Knox 2019). Linking activities in TE with placement in relation to PDC is important (Baran et al. 2019; Instefjord and Munthe 2017) also when it comes to aspects of digital citizenship (cf. Gudmundsdottir and Hatlevik 2020), and future studies could explore this further both in Sweden and other national contexts.

This paper also identifies challenges relating to how TE is organized locally, such as distribution of course responsibility, which could result in key areas of Core Education Subjects being overlooked. Because questions relating to digital citizenship are considered important, many speculate that other TEDs address such questions since they themselves do not, which echoes the need for a review of course content and program structure. Self-image adds further complexity as some TEDs describe how they contribute in ways linked to their work in other fields or that students know more than they do, hence addressing digital technologies becomes the responsibility of colleagues with a background in education. This could also be interpreted as a self-perceived lack of PDC because, being part of a team, it becomes easier to opt out and rely on colleagues to address digital technologies, thus increasing one’s sense of agency as a professional (Roumbanis-Viberg et al. 2021).

Focusing on a module on democracy and education mandatory for all subject student teachers, this paper has highlighted the need for support through TEDs’ professional development and national policy. Digital citizenship is a growing research field (Richardson et al. 2021) which has become increasingly international, and the European Commission (2021) lists digital citizenship among the 'Digital Decade' targets for 2030. Although the context of this paper is Sweden, the results are likely relevant for consideration in other national contexts, for instance, where there is ambiguity as to central guidelines on digital citizenship in education. Also, the results could contribute to theory on digital citizenship and PDC in TE by adding to an expanding body of research called for by previous studies (e.g., Christensen et al. 2021; Lauricella et al. 2020; Uertz et al. 2018).

Further adding to the contributions of this paper could be the exploration of digital citizenship in TE focusing on the school subject social studies, which historically has carried the responsibility explicitly and implicitly for citizenship formation in Sweden (Englund 1986/2005). This could be reflected by TEDs’ speculations that questions relating to digital citizenship are addressed in social studies TE. Future studies could also explore TEDs’ PDC in educational practices (cf. Uertz et al. 2018) specifically in relation to digital citizenship. Cross-national studies could contribute by focusing, for instance, on the European context using digital competence frameworks for citizens developed by the European Commission, or the Nordic countries given their common approach to digital competence.

## Limitations

This study has several limitations. Firstly, this study draws on data from seven out of the main 25 TEIs that prepare subject teachers in Sweden, and while TEDs’ views as discussed in this paper are likely common at other TEIs given the number of participating institutions, such conclusions cannot be drawn from present data. Secondly, the focus on Swedish TE entails other considerations. Questions relating to digital citizenship might be discussed elsewhere in subject TE, but as the democratic assignment concerns all teachers, the mandatory E&D module is a key area in preparing student teachers for democratic work. This suggests that translations to other national contexts may be aided by focusing specifically on TE courses or modules on democratic work. Similarly, almost half of the TEDs in this study have a background in fields other than education which is not uncommon in Sweden, and while they have extensive TE teaching experience, this may be important when considering the results in other contexts. Thirdly, as the purpose of the study is to explore TEDs’ views, present data are insufficient for TEI comparisons or deducing the extent to which decisions on teaching priorities are made by individuals, such as TEDs or course directors, or made elsewhere, for example, in TED teams or at the department level. Lastly, at the time of the study, the first batches of Covid-19 vaccines were distributed, and anti-vaccine campaigns were spreading, for instance, through large protests and on social media, which may have contributed to participants’ associating digital citizenship with source criticism. Also, some participants had not reflected on questions relating to digital citizenship in TE previously, and in the absence of central guidelines on digital citizenship, this may explain to some degree the presence of normative approaches in TEDs’ conceptualizations (cf. Davis 2020).
